# Patients with complex and very-early-onset *ATL1*-related spastic paraplegia offer insights on genotype/phenotype correlations and support for autosomal recessive forms of SPG3A

**DOI:** 10.1007/s00415-024-12565-0

**Published:** 2024-07-13

**Authors:** Angélique Hamamie-Chaar, Mathilde Renaud, Pinar Gençpinar, Ange-Line Bruel, Christophe Philippe, Julien Maraval, Caroline Racine, Nawale Hadouiri, Laetitia Lambert, Emmanuelle Schmitt, Guillaume Banneau, Armand Hocquel, Christel Thauvin-Robinet, Laurence Faivre, Quentin Thomas

**Affiliations:** 1 Department of Clinical Genetics, Dijon University Hospital, Dijon, France; 2 Department of Clinical Genetics, CHRU Nancy, Nancy, France; 3 INSERM-U1256 NGERE, Université de Lorraine, Nancy, France; 4 Department of Pediatric Neurology, İzmir Katip Çelebi University, Izmir, Turkey; 5 Inserm UMR1231 Team GAD, University of Burgundy and Franche-Comté, Dijon, France; 6 Department of Clinical Genetics, CHU Toulouse, Toulouse, France; 7 Pitié-Salpêtrière, Department of Genetics, Sorbonne Université, AP-HP, Paris, France

**Keywords:** Spastic paraplegia, ATL1, SPG3A, Early-onset, Homozygous, Biallelic, Loss-of-function

## Abstract

Spastic paraplegia type 3A (SPG3A) is the second most common form of hereditary spastic paraplegia (HSP). This autosomal-dominant-inherited motor disorder is caused by heterozygous variants in the *ATL1* gene which usually presents as a pure childhood-onset spastic paraplegia. Affected individuals present muscle weakness and spasticity in the lower limbs, with symptom onset in the first decade of life. Individuals with SPG3A typically present a slow progression and remain ambulatory throughout their life. Here we report three unrelated individuals presenting with very-early-onset (before 7 months) complex, and severe HSP phenotypes (axial hypotonia, spastic quadriplegia, dystonia, seizures and intellectual disability). For 2 of the 3 patients, these phenotypes led to the initial diagnosis of cerebral palsy (CP). These individuals carried novel *ATL1* pathogenic variants (a de novo* ATL1* missense p.(Lys406Glu), a homozygous frameshift p.(Arg403Glufs*3) and a homozygous missense variant (p.Tyr367His)). The parents carrying the heterozygous frameshift and missense variants were asymptomatic. Through these observations, we increase the knowledge on genotype–phenotype correlations in SPG3A and offer additional proof for possible autosomal recessive forms of SPG3A, while raising awareness on these exceptional phenotypes. Their ability to mimic CP also implies that genetic testing should be considered for patients with atypical forms of CP, given the implications for genetic counseling.

## Introduction

Hereditary spastic paraplegias (HSP) are a clinically and genetically heterogeneous group of neurodegenerative diseases characterized by the common hallmark of progressive spasticity and weakness of the lower limbs [1]. More than 80 spastic paraplegia genes (SPG) have been identified over the last decades for which autosomal dominant, recessive, X-linked and mitochondrial modes of transmissions have been reported [2]. Among them, SPG3A [MIM:182600] is the second most frequent autosomal dominant HSP, and the most frequent form of early-onset autosomal dominant HSP [3, 4]. SPG3A is usually caused by heterozygous missense variants in the *ATL1* gene which encodes the atlastin protein, generating a dominant-negative effect [5, 6]. This GTPase is mainly expressed in brain tissues, is located in the Golgi apparatus and the endoplasmic reticulum, and interacts with spastin and mitogen-activated protein kinase 4 [7]. It is involved in endoplasmic reticulum tubular network biogenesis and axonal maintenance [8]. SPG3A is characterized by an early age of onset, typically before the end of the first decade, with a mean age at first symptoms of 4.6 years, and presents with a “pure” phenotype, meaning that other neurological syndromes such as cerebellar ataxia or developmental delay are normally not observed [3]. Gait instability and lower limbs stiffness usually appear first, with a slow progression, allowing most patients to maintain an independent ambulation throughout their life.

Besides this historical phenotype, rare reports have been made of SPG3A patients with complex phenotypes which included neuropathy, cognitive impairment, dysphagia, dysarthria or limb amyotrophy [9, 10]. Few articles have also reported patients carrying biallelic *ATL1* variants who presented with an earlier and more severe phenotype, while heterozygous parents were asymptomatic, raising the question of possible autosomal recessive forms of SPG3A [11, 12]. Additional reports are however needed to sustain this statement.

Here, we report three children with very early onset, complex and severe phenotypes who started displaying symptoms in their first months of life. We discuss a possible increase in knowledge on genotype–phenotype correlations in SPG3A, extend SPG3A’s phenotype, and offer additional proof for possible autosomal recessive forms of SPG3A.

## Methods

Patient’s 1 variant was identified through exome sequencing, performed as detailed in previous publications [13]. Following this identification, a call for collaboration and data sharing was initiated in France through the AnDDI-Rares network and the national network of expert centers for neurogenetic disorders, and in Europe though the European Reference Network ITHACA. The AnDDI-Rares collaboration call identified patient 2 and the ITHACA collaboration call patient 3, both diagnosed by gene panel sequencing. Within the ninety-six patients with confirmed pathogenic *ATL1* variants in the French neurogenetic databases, none had a severe phenotype evidenced in the first year of life.

## Results

### Patient 1

The first patient was a 6-year-old male referred to our center regarding spastic quadriplegia, dystonic movements and axial hypotonia. He was the first born of healthy unrelated parents. After an uneventful pregnancy, he was delivered at full-term without any complications. Birth measurements and APGAR scores were within norms. Examination at birth revealed axial hypotonia.

At 3 months, he started presenting progressive hypertonia of the four limbs and could not hold his head. Just before 2 years, he started presenting dystonia of his four limbs for which L-Dopa was administered, without significant improvement. Clinical examination also revealed impaired gross motor skills with difficult prehension, inability to hold his head and to sit alone. At 8 years, he still could not hold his head, roll over, sit or walk unaided. He presented several musculoskeletal deformities which included asymmetric equinovarus, kyphosis, scoliosis and hip dislocation. Limb spasticity altered his fine hand movements and generated a typical flexion of elbows and wrists. Due to chewing difficulties, he was only fed on soft food. Facial hypotonia generated drooling and made speech impossible. He could however communicate through augmentative and alternative communication (AAC). He was wheelchair-bound and could operate his power wheelchair himself with head array. This ability to communicate through AAC and to steer his wheelchair, added to the fact that he could read small sentences and do simple math, contrasted dramatically with his impaired motor development. Brain MRI at 3 years found a temporal subarachnoid cyst with mild and diffuse parietal white matter T2 hyperintensities.

This clinical presentation led to the initial diagnosis of cerebral palsy (CP). However, over the years the lack of congruent medical history and brain MRI abnormalities evocative of CP motivated additional tests. Biological investigations including lactate, thyroid hormone, plasmatic amino acid chromatography and urinalysis were normal. Cerebrospinal fluid analyses (protein, cells, glucose, neurotransmitters) were also normal. Array-CGH and a targeted panel sequencing of 450 genes associated with intellectual disability failed to identify any pathogenic variant. Exome sequencing revealed a heterozygous de novo* ATL1* variant (NM_015915.4.c:1216A > G; p.Lys406Glu). This missense was absent from general population databases (gnomAD v3.1.2) and presented in silico scores in favor of its pathogenicity (CADD5: 21.9; PolyPhen-: 0.68–0.79; GERP: 5.46; grantham: 56, misZ: 2.809). It affected an amino acid residue located in the 3HB domain of the ATL1 protein. According to the ACMG classification, this missense was classified as pathogenic (PS2, PM1, PM2, PM5, PP3, PP5).

### Patient 2

The second patient was a 7-year-old male who presented with severe spastic paraplegia and intellectual disability. He was the first child of a related couple from Turkey (parents were first cousins). He was born full-term with APGAR scores of 2/8, due to circular cord. Birth weight and height were normal but head circumference was below the 10th percentile (32.5 cm). Examination at birth was marked with axial hypotonia. At 9 months, he presented a generalized tonic–clonic seizure requiring antiepileptic drugs (sodium valproate). Neurological examination revealed lower limb spasticity, in addition to the already observed axial hypotonia. Upon clinical follow-up at 2 years, head control had been acquired but he could not sit or stand and presented language delay with absent speech. Lower limbs spasticity worsened resulting in spastic diplegia. At 4 years head circumference was below the 3rd percentile, sitting was still unsteady, and walking was only possible for a few steps with human help. He could say a few words but could not associate them. Dysarthria due to facial hypotonia was noted, along with mild swallowing impairment. At 7 years, he presented severe spastic diplegia. Upper limbs did not show spasticity but the patient had impaired fine hand movements. Clinical assessment of his functional abilities was in favor of a moderate intellectual disability requiring institutionalization in a center dedicated for disabled children.

**Fig. 1:**
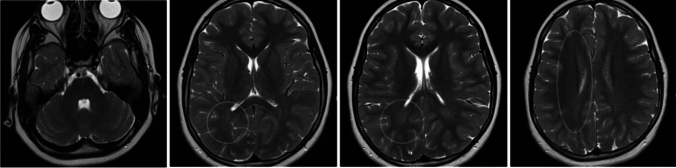
Patient 2’s brain MRI at the age of 5. Notice the posterior T2 white matter hyperintensities evocative of myelination delay (white circles)

Similarly to patient 1, this individual was first diagnosed with CP until follow-up in an expert center and the lack of congruent brain MRI abnormalities motivated further tests. Indeed, brain MRI at 5 years revealed white matter T2 hyperintensities evocative of myelination delay (Fig. 1). Laboratory exams including blood and urinary tests were normal. A targeted panel sequencing of HSP genes was done and revealed a novel homozygous *ATL1* frameshift variant (NM_015915.4:c.1207del; p.Arg403Glufs*3, exon 12/14). This variant most likely activates the nonsense-mediated mRNA decay surveillance pathway, thus resulting in the absence of the atlastin protein. According to the ACMG classification, this variant was classified as pathogenic (PVS1, PP5, PM2). Familial segregation revealed that both parents were asymptomatic heterozygous carriers.

### Patient 3

This third patient was a 10-year-old male who started displaying global hypotonia at the age of 3 months, followed by spasticity of the four limbs at the age of 7 months. Follow-up confirmed the phenotype of spastic tetraplegia, secondarily associated with global developmental delay, as he never acquired the ability to sit, walk, or talk. Upon last follow-up, he could only utter isolated words. Brain MRI performed at the age of 2 years was normal. Similarly to previous patients, he also presented with swallowing problems and dysarthria. A panel of genes associated with HSP was sequenced and revealed a homozygous missense variation within exon 11 of the *ATL1* gene (c.1099T > C; (p.Tyr367His). Familial segregation revealed both parents, who were not related but originated from the same small village, were asymptomatic heterozygous carriers. Here again in silico scores pleaded in favor of the variant’s pathogenicity and the variant impacted a highly conserved amino acid, within the 3HB domain. However, the fact that the parents were asymptomatic heterozygous carriers, evocative of the rarely described and poorly known autosomal recessive mode of inheritance, led to the variation being classified as of “unknown significance” according to the ACMG criteria.

## Discussion

We report 3 unrelated patients presenting with neonatal, complex and severe phenotypes of SPG3A. Their very early age of onset, the severity and the wide range of symptoms they presented clearly contrasted with the usual phenotype of SPG3A patients. Two of the 3 patients (patients 2 & 3) even presented with confirmed intellectual disability, independently from their motor impairment. All patients presented the striking sequence of an initial axial hypotonia, secondarily associated with progressive limb spasticity. This feature has, to our knowledge, never been reported in SPG3A and is also unusual in childhood or adult-onset HSP. It may thus represent an extension of SPG3A’s phenotype, only observed in neonatal forms.

Such severe presentations are extremely rare in SPG3A as only 5 reports have been made of individuals with such early (first symptoms before 6 months of age), severe and complex phenotypes [9, 10, 14, 15] (Table 1). These individuals presented phenotypes very congruent to the ones of our patients with severe spastic tetraplegia, but also difficulties in swallowing, chewing or speaking (Table 1).

**Table 1:** Clinical characteristics of our patients along with previously published cases of severe and/or complicated forms of SPG3A

Reference	Haberlová [10]	Fusco [9]	Yonekawa [15]	Kelly et al. [16]	Alecu et al. [18]	Patient 1	Patient 2	Patient 3
Age (years)	7	17	12	15	Between 2 and 15	6	7	10
Sex	M	F	M	M	3M–2F	M	M	M
Age at onset	3 m	3 m	< 6 m	< 6 m	All before 9 m, 2 at 1 month	< 3 month	9 month	3 month
Symptom at onset	Motor developmental delay	Axial hypotonia	Lower limb spasticity	Motor developmental delay	Neonatal ankle clonus (P2), lower limb stiffness (P5), delayed motor development (P1, 3, 4)	Global stiffness and axial hypotonia	Axial hypotonia and lower limb spasticity	Axial hypotonia and limbs spasticity
Phenotype	Severe spastic tetraplegia	Complex severe spastic tetraplegia	Severe spastic tetraplegia	Complex severe spastic tetraplegia	Complex severe spastic paraplegia	Dystonia, spastic tetraplegia	Spastic paraplegia	Complex severe spastic tetraplegia
Maximal motor function	Sit and raise himself onto his knees	Unable to sit or walk	Unable to sit or walk	Unable to sit or walk		Unable to sit or walk	Sit, unable to walk without human help	Unable to sit, walk or talk
Fine hand movement	Severely impaired	NA	Severely impaired	Severely impaired	Impaired	Severely impaired	Impaired	impaired
Cranial nerve abnormalities	Severe dysphagia and dysarthria	Dysphagia, dysmasesis	Dysarthria, dysphagia, dysmasesis	Swallowing problems	Dysarthria (3/5)	Swallowing problems, dysarthria	Facial hypotonia	Swallowing problems, dysarthria
Cognitive impairment	Normal nonverbal intellect	Moderate intellectual disability	Normal nonverbal intellect	Normal nonverbal intellect	Developmental delay	Normal nonverbal intellect	Intellectual disability	
*ATL1* variant c. (NM_015915.4) p.	de novo c.1223T > C p.Met408Thr	de novo c.1193C > T p.Ser398Phe	de novo c.1226G > A p.Gly409Asp	de novo c.1248_1249insAAT p.Arg416_Tyr417insAsn	de novo p.Ser160Pro p.Asn252Lys p.Lys407del p.Lys407Met p.Met408Thr	de novo c.1216A > G p.Lys406Glu	Homozygous c.1207delC p.Arg403Glufs*3	Homozygous c.1099T > C (p.Tyr367His)
Brain and spinal MRI	“Unspecific periventricular gliosis” Normal spinal MRI	Normal	Normal	Thin corpus callosum	Thinning of the corpus callosum and reduced white matter volume (2/5)	Mild and diffuse parietal subcortical white matter hyperintensities	T2 periventricular parietal white matter hyperintensities, myelination delay	Normal

*M* Male; *F* female; *m* months; *P* proband, *NA* not available

Over the years, no clear genotype/phenotype correlation has been demonstrated in SPG3A. It is however noteworthy that these severe and early-onset phenotypes have been particularly associated with missense variants within the 3HB domain (between the 347th and 432nd amino acids) and the linker region [16]. Three-dimensional structural studies of large SPG3A cohorts have shown that these clusters may be explained by the importance of some specific amino acids in the protein’s ability to form monomers [17]. Indeed atlastin-1 functions by forming tetrameric complexes and formation of heterocomplexes between normal and abnormal atlastin-1 may interfere with tetramer activity [8]. In this process, some residues carry a more critical role in the crossover dimerization, and may thus impact more significantly the protein’s function when altered by a variation.

Our report reinforces these observations, especially patients 1 and 3 whose variants (p.Lys406Glu, (p.Tyr367His)) were missense variants located in the 3HB domain, very close to the previously reported p.Met408Thr and p.Gly409Asp variants of other early-onset forms.

Regarding patient 2, he harbored a homozygous frameshift variant (p.Arg403Glufs*3). Very few reports have been made of patients with biallelic *ALT1* variants [11, 12] and even fewer of loss-of-function variants [18]. Willkomm et al*.* described such a case: an Arabic consanguineous family in which three of the six siblings had HSP [12]. The three brothers were homozygous for a truncating *ATL1* variant (p.Arg217*). First symptoms occurred between 18 months and 5 years of age with lower limb spasticity. They had learning difficulties and slow speech. In this family, heterozygous carriers were asymptomatic, as observed in our patients’ families. Frameshift and stop-gain variants result in mRNAs harboring a premature termination codon, leading to their degradation via the NMD system. Consequently, individuals carrying biallelic *ATL1* truncating variants such as patient 2 most likely present a complete lack of atlastin-1 protein, thus explaining their very severe, early onset and complex phenotypes. On the other hand, individuals with a heterozygous truncating variant do not produce any abnormal atlastin-1 protein. The atlastin-1 protein produced from their normal allele is indeed functional and can dimerize correctly, most likely explaining why such individuals are asymptomatic, and why SPG3A presents as an autosomal recessive disorder in such cases.

The mechanisms that explain why some missense variants are only pathogenic at the heterozygous state is however more elusive. Beyond the 2 patients we report here (1 and 3), Khan et al*.* reported a consanguineous Pakistani family in which six males carried a homozygous *ATL1* missense variant (p.Arg118Gln) who had a pure HSP with an onset before 2 years of age. Altogether these observations, support the statement that missense variants may also be associated with autosomal recessive forms of SPG3A.

Finally, it is worthy to note that patients 1 and 2 were both temporarily diagnosed with Cerebral Palsy (CP). The diagnosis of CP may be made challenging by its wide and heterogenous range of clinical manifestations which includes spastic diplegia, and developmental delay. Numerous genetic disorders, including HSP, may thus mimic CP, especially because a normal brain MRI does not necessarily rule out CP [19–21]. Yet, identification of a genetic, hereditary disorder is critical to offer adequate care, follow-up and genetic counseling. In the case of our patients, investigations were continued because their medical history lacked perinatal brain injury, and because brain MRIs showed abnormalities inconsistent with what is normally observed in CP (namely basal ganglia T2 FLAIR hyperintensities in the acute phase, and signs of Wallerian degeneration with cortical atrophy at the chronic stage).

In conclusion, we report here the cases of 3 patients with severe forms of early-onset SPG3A, offering detailed descriptions of this poorly known phenotype made of neonatal hypotonia, secondary limbs spasticity (spastic para or tetraplegia), developmental delay, speech and swallowing problems. We also show that these patients may present T2 white matter hyperintensities evocative of myelination delay. We finally provide knowledge on the molecular insights of biallelic variants, genotype/phenotype correlations and further evidence for an autosomal recessive form of *ATL1*-related HSP.

## Data Availability

All data relevant to this study have been disclosed in the manuscript. Further information may be shared upon request.

## References

[1] Salinas S, Proukakis C, Crosby A, Warner TT (2008) Hereditary spastic paraplegia: clinical features and pathogenetic mechanisms. Lancet Neurol 7:1127–1138. 10.1016/S1474-4422(08)70258-8 19007737 10.1016/S1474-4422(08)70258-8

[2] Finsterer J, Löscher W, Quasthoff S, Wanschitz J, Auer-Grumbach M, Stevanin G (2012) Hereditary spastic paraplegias with autosomal dominant, recessive, X-linked, or maternal trait of inheritance. J Neurol Sci 318:1–18. 10.1016/J.JNS.2012.03.025 22554690 10.1016/J.JNS.2012.03.025

[3] Namekawa M, Ribai P, Nelson I, Forlani S, Fellmann F, Goizet C et al (2006) SPG3A is the most frequent cause of hereditary spastic paraplegia with onset before age 10 years. Neurology 66:112–114. 10.1212/01.WNL.0000191390.20564.8E 16401858 10.1212/01.WNL.0000191390.20564.8E

[4] Dürr A, Camuzat A, Colin E, Tallaksen C, Hannequin D, Coutinho P et al (2004) Atlastin1 mutations are frequent in young-onset autosomal dominant spastic paraplegia. Arch Neurol 61:1867–1872. 10.1001/ARCHNEUR.61.12.1867 15596607 10.1001/ARCHNEUR.61.12.1867

[5] Ulengin I, Park JJ, Lee TH (2015) ER network formation and membrane fusion by atlastin1/SPG3A disease variants. Mol Biol Cell 26:1616–1628. 10.1091/MBC.E14-10-1447 25761634 10.1091/MBC.E14-10-1447 PMC4436774

[6] Álvarez V, Sánchez-Ferrero E, Beetz C, Díaz M, Alonso B, Corao AI et al (2010) Mutational spectrum of the SPG4 (SPAST) and SPG3A (ATL1) genes in Spanish patients with hereditary spastic paraplegia. BMC Neurol. 10.1186/1471-2377-10-89 20932283 10.1186/1471-2377-10-89 PMC2964648

[7] Wang S, Tukachinsky H, Romano FB, Rapoport TA (2016) Cooperation of the ER-shaping proteins atlastin, lunapark, and reticulons to generate a tubular membrane network. Elife. 10.7554/ELIFE.18605 27619977 10.7554/ELIFE.18605 PMC5021524

[8] Zhu PP, Patterson A, Lavoie B, Stadler J, Shoeb M, Patel R et al (2003) Cellular localization, oligomerization, and membrane association of the hereditary spastic paraplegia 3A (SPG3A) protein atlastin. J Biol Chem 278:49063–49071. 10.1074/JBC.M306702200 14506257 10.1074/JBC.M306702200

[9] Fusco C, Frattini D, Farnetti E, Nicoli D, Casali B, Della GE (2012) Very early onset and severe complicated phenotype caused by a new spastic paraplegia 3A gene mutation. J Child Neurol 27:1348–1350. 10.1177/0883073811435245 22378671 10.1177/0883073811435245

[10] Haberlová J, Claeys KG, Zámečník J, De Jonghe P, Seeman P (2008) Extending the clinical spectrum of SPG3A mutations to a very severe and very early complicated phenotype. J Neurol 255:927–928. 10.1007/S00415-008-0598-Z 18446315 10.1007/S00415-008-0598-Z

[11] Khan TN, Klar J, Tariq M, Anjum Baig S, Malik NA, Yousaf R et al (2014) Evidence for autosomal recessive inheritance in SPG3A caused by homozygosity for a novel ATL1 missense mutation. Eur J Hum Genet 22:1180–1184. 10.1038/EJHG.2014.5 24473461 10.1038/EJHG.2014.5 PMC4169543

[12] Willkomm L, Heredia R, Hoffmann K, Wang H, Voit T, Hoffman EP et al (2016) Homozygous mutation in Atlastin GTPase 1 causes recessive hereditary spastic paraplegia. J Hum Genet 61:571–573. 10.1038/JHG.2016.6 26888483 10.1038/JHG.2016.6

[13] Thomas Q, Vitobello A, Tran Mau-Them F, Duffourd Y, Fromont A, Giroud M et al (2020) High efficiency and clinical relevance of exome sequencing in the daily practice of neurogenetics. J Med Genet. 10.1136/jmedgenet-2020-107369 34085946 10.1136/jmedgenet-2020-107369

[14] Morais S, Raymond L, Mairey M, Coutinho P, Brandão E, Ribeiro P et al (2017) Massive sequencing of 70 genes reveals a myriad of missing genes or mechanisms to be uncovered in hereditary spastic paraplegias. Eur J Hum Genet 25:1217–1228. 10.1038/EJHG.2017.124 28832565 10.1038/EJHG.2017.124 PMC5643959

[15] Yonekawa T, Oya Y, Higuchi Y, Hashiguchi A, Takashima H, Sugai K et al (2014) Extremely severe complicated spastic paraplegia 3A with neonatal onset. Pediatr Neurol 51:726–729. 10.1016/J.PEDIATRNEUROL.2014.07.027 25193411 10.1016/J.PEDIATRNEUROL.2014.07.027

[16] Kelly CM, Zeiger PJ, Narayanan V, Ramsey K, Sondermann H (2022) A novel insertion mutation in atlastin 1 is associated with spastic quadriplegia, increased membrane tethering, and aberrant conformational switching. J Biol Chem. 10.1016/j.jbc.2021.101438 34808209 10.1016/j.jbc.2021.101438 PMC8688574

[17] Bian X, Klemm RW, Liu TY, Zhang M, Sun S, Sui X et al (2011) Structures of the atlastin GTPase provide insight into homotypic fusion of endoplasmic reticulum membranes. Proc Natl Acad Sci USA 108:3976–3981. 10.1073/PNAS.1101643108/-/DCSUPPLEMENTAL/PNAS.201101643SI.PDF 21368113 10.1073/PNAS.1101643108/-/DCSUPPLEMENTAL/PNAS.201101643SI.PDF PMC3054032

[18] Alecu JE, Saffari A, Jordan C, Srivastava S, Blackstone C, Ebrahimi-Fakhari D (2023) De novo variants cause complex symptoms in HSP-ATL1 (SPG3A) and uncover genotype-phenotype correlations. Hum Mol Genet 32:93–103. 10.1093/HMG/DDAC182 35925862 10.1093/HMG/DDAC182 PMC9838092

[19] Himmelmann K, Horber V, Sellier E, De la Cruz J, Papavasiliou A, Krägeloh-Mann I (2021) Neuroimaging patterns and function in cerebral palsy-application of an MRI classification. Front Neurol. 10.3389/FNEUR.2020.617740 33613420 10.3389/FNEUR.2020.617740 PMC7887285

[20] Novak I, Morgan C, Adde L, Blackman J, Boyd RN, Brunstrom-Hernandez J et al (2017) Early, accurate diagnosis and early intervention in cerebral palsy: advances in diagnosis and treatment. JAMA Pediatr 171:897–907. 10.1001/JAMAPEDIATRICS.2017.1689 28715518 10.1001/JAMAPEDIATRICS.2017.1689 PMC9641643

[21] Pearson TS, Pons R, Ghaoui R, Sue CM (2019) Genetic mimics of cerebral palsy. Mov Disord 34:625–636. 10.1002/MDS.27655 30913345 10.1002/MDS.27655

